# Comparison of Four Carbapenemase Detection Methods for *bla*_KPC-2_ Variants

**DOI:** 10.1128/Spectrum.00954-21

**Published:** 2021-12-22

**Authors:** Li Ding, Qingyu Shi, Renru Han, Dandan Yin, Shi Wu, Yang Yang, Yan Guo, Demei Zhu, Fupin Hu

**Affiliations:** a Institute of Antibiotics, Huashan Hospital, Fudan University, Shanghai, People's Republic of China; b Key Laboratory of Clinical Pharmacology of Antibiotics, Ministry of Health, Shanghai, People's Republic of China; Labcorp

**Keywords:** *bla*_KPC-2_ variants, mCIM, carbapenemase inhibitor enhancement method, NG-test Carba 5, GeneXpert Carba-R

## Abstract

Recently, various *bla*_KPC-2_ variants resistant to ceftazidime-avibactam have begun to emerge in clinical settings, but it is unclear which testing method is most appropriate for detecting these variants. Strains were subjected to antimicrobial susceptibility testing using the broth microdilution method. Four carbapenemase detection methods, modified carbapenem inactivation method (mCIM) and EDTA carbapenem inactivation method (eCIM), APB/EDTA (carbapenemase inhibitor APB [3-aminophenylboronic acid] and EDTA enhancement method), NG-test Carba 5, and GeneXpert Carba-R were used to try to detect KPC-2 variants in 19 Klebsiella pneumoniae isolates. Among those *bla*_KPC-2_ variants, *bla*_KPC-33_-, *bla*_KPC-35_-, *bla*_KPC-71_-, *bla*_KPC-76_-, *bla*_KPC-78_-, and *bla*_KPC-79_-positive isolates accounted for 26.3% (5/19), 15.8% (3/19), 5.3% (1/19), % 42.1% (8/19), 5.3% (1/19), and 5.3% (1/19), respectively. All 19 K. pneumoniae carrying *bla*_KPC-2_ variants showed resistance to ceftazidime-avibactam (MICs:16 to >64 mg/L), and 14 strains were susceptible to imipenem (MICs: 0.25 to 1 mg/L). None of the *bla*_KPC-2_ variants could be detected using either the mCIM or the APB/EDTA method, while five strains carrying *bla*_KPC-2_ variants (*bla*_KPC-35_, *bla*_KPC-78_, and *bla*_KPC-79_) tested KPC positive when using NG-test Carba 5. However, GeneXpert Carba-R was able to detect *bla*_KPC-2_ variants (harboring *bla*_KPC-33_, *bla*_KPC-35_, *bla*_KPC-71_, *bla*_KPC-76_, *bla*_KPC-78_, and *bla*_KPC-79_) carried by all 19 K. pneumoniae. The emergence of new KPC variants poses an increased challenge for carbapenemase detection methods, and laboratories should use the appropriate assays to accurately detect these variants.

**IMPORTANCE** Carbapenemase detection is essential for the appropriate treatment of CRE infections. Several clinical laboratories have begun using relevant carbapenemase assays such as mCIM and eCIM, the APB/EDTA method, NG-test Carba 5, and GeneXpert Carba-R to detect carbapenemases. Nevertheless, some of these methods may have limitations for detecting *bla*_KPC-2_ variants. Additionally, there has been little relevant research on evaluate the differences between these standard methods for detecting *bla*_KPC-2_ variants. Therefore, we investigated the reliability of these classic methods for assessing 19 K. pneumoniae with *bla*_KPC-2_ variants. Our results showed that none of the *bla*_KPC-2_ variants could be detected using either the mCIM or APB/EDTA method, while five strains (harboring *bla*_KPC-35_, *bla*_KPC-78_,and *bla*_KPC-79_) tested KPC positive when using NG-test Carba 5. GeneXpert Carba-R could detect six *bla*_KPC-2_ variants carried by all 19 K. pneumoniae. This study may be valuable for clinical laboratories in their efforts to test for various *bla*_KPC-2_ variants.

## INTRODUCTION

Carbapenem-resistant *Enterobacterales* (CRE) are a significant concern for patients in health care facilities. Some bacteria in this family are resistant to nearly all β-lactams and other antibacterial agents, leaving more toxic or less effective treatment options (1, 2). Studies have shown that clinical treatment failure and mortality rates are 2 to 3 times higher in CRE-infected patients than in CSE (carbapenem-susceptible *Enterobacterales*)-infected patients (3, 4). Carbapenemase production is the predominant mechanism of resistance in CRE, especially Klebsiella pneumoniae carbapenemases (KPC) (5 to 7). An extensive multicenter survey in China revealed that 70.3% (307/437) of CRE isolated from adult patients were KPC-2-producers (8).

New β-lactam-β-lactamase inhibitor combinations have been developed to cope with the infection challenge caused by KPC-producing *Enterobacterales*, including ceftazidime-avibactam, meropenem-vaborbactam, and imipenem-relebactam (9–11). However, with the clinical application of these drugs, KPC-producing strains have mutated to adapt to the pressure of new antibiotics, generating new *bla*_KPC_ subtypes, such as *bla*_KPC-14_, *bla*_KPC-28_, and *bla*_KPC-33_, based on mutations in *bla*_KPC-2_ or *bla*_KPC-3_ (11, 12). So far (as of July 2021), 88 KPC alleles have been uploaded to the NCBI database (13). The most critical phenotypic features of KPC variants are their resistance to ceftazidime-avibactam and their restoration of susceptibility to meropenem or imipenem, which is mainly due to amino acid substitutions and conformational changes in the carbapenemase active site (11–14).

Current methods for detecting carbapenemases include the modified carbapenem inactivation method (mCIM) and EDTA carbapenem inactivation method (eCIM), the carbapenemase inhibitor APB (3-aminophenylboronic acid) and EDTA enhancement method (APB/EDTA method), the lateral flow immunochromatographic assay (LFIA) NG-test Carba 5, and the automated real-time quantitative PCR (RT-qPCR)-based GeneXpert Carba-R (15–17). However, there has been little relevant research to evaluate the differences between these standard methods in detecting *bla*_KPC-2_ variants. Therefore, in this study, we investigated the reliability of these classic methods for assessing *bla*_KPC-2_ variants.

## RESULTS

### Antimicrobial susceptibility testing.

As shown in Table 1 and Table 2, the susceptibility phenotypes of 19 strains carrying the *bla*_KPC-2_ variant were relatively similar. All *bla*_KPC-2_ variant-producing K. pneumoniae were resistant to ceftazidime-avibactam with a MIC range from 16 mg/L to >64 mg/L, and 18 strains showed resistance to ertapenem (MICs: 16 to 32 mg/L), except for one *bla*_KPC-35_ positive K. pneumoniae (no. 8) which appeared to be sensitive to ertapenem; this may be related to the slow growth and small colony morphology of this strain. Interestingly, 14 isolates carrying the *bla*_KPC-2_ variant were susceptible to imipenem (MICs: 0.25 to 1 mg/L), while the remaining strains (four strains harboring *bla*_KPC-76_, one strain harboring *bla*_KPC-79_) were intermediate or resistant to imipenem (MICs: 2 to 4 mg/L). Five strains were susceptible to meropenem (MICs: 1 mg/L) while the remaining strains were intermediate or resistant to meropenem (MICs: 2 to 8 mg/L). In addition, all strains showed sensitivity to meropenem-vaborbactam (MICs:0.03 to 4 mg/L).

**TABLE 1:** Characterization of the relevant antimicrobial sensitivity profiles of *bla*_KPC-2_ variants^*a*^

No.	Specimen date	Source	KPC variant	Nucleotide	Amino acid substitution	Other β-lactam genes	MIC (mg/L)					Detection method				
							ETP	IPM	MEM	CZA	MEV	mCIM	eCIM	APB/EDTA	NG-test Carba 5	GeneXpert Carba-R
1	2019/12	UR	KPC-33	G532T	D179Y	ND	32 (R)	0.5 (S)	4 (R)	>64 (R)	2(S)	–		–	–	KPC+
2	2020/05	UR	KPC-33	G532T	D179Y	ND	16 (R)	0.5 (S)	1 (S)	64 (R)	2(S)	–		–	–	KPC+
3	2020/10	SF	KPC-33	G532T	D179Y	*bla*_LAP-2_, *bla*_SHV-12_, *bla*_TEM-1B_	32 (R)	0.5 (S)	2 (I)	>64 (R)	1(S)	–		–	–	KPC+
4	2020/09	SP	KPC-33	G532T	D179Y	ND	16 (R)	0.25 (S)	1 (S)	64 (R)	1(S)	–		–	–	KPC+
5	2020/09	MB	KPC-33	G532T	D179Y	ND	16 (R)	0.5 (S)	2 (I)	>64 (R)	1(S)	–		–	–	KPC+
6	2020/09	SF	KPC-35	T503C	L169P	*bla*_LAP-2_, *bla*_SHV-12_, *bla*_TEM-1B_	16 (R)	1 (S)	4 (R)	64 (R)	2(S)	–		–	KPC+	KPC+
7	2020/09	SP	KPC-35	T503C	L169P	*bla*_LAP-2_, *bla*_SHV-12_, *bla*_TEM-1B_	8 (R)	0.25 (S)	1 (S)	32 (R)	0.5(S)	–		–	KPC+	KPC+
8	2020/09	AB	KPC-35	T503C	L169P	*bla*_TEM-1B_, *bla*_SHV-12_	0.125 (S)	1 (S)	1 (S)	16 (R)	0.03(S)	–		–	KPC+	KPC+
9	2021/03	SP	KPC-71	ACT insertion	181S_182P insertion	*bla*_LAP-2_, *bla*_SHV-12_, *bla*_TEM-1B,_ *bla*_CTX-M-65_	16 (R)	0.5 (S)	2 (I)	>64 (R)	0.5(S)	–		–	–	KPC+
10	2020/01	UR	KPC-76	G532T+TR insertion	D179Y+262V_268N dup	*bla*_SHV-12_, *bla*_CTX-M-65_, *bla*_TEM-1_, *bla*_DHA-1_, *bla*_LAP-2_	32 (R)	2 (I)	4 (R)	>64 (R)	2(S)	–		–	–	KPC+
11	2020/03	SP	KPC-76	G532T+TR insertion	D179Y+262V_268N dup	*bla*_SHV-12_, *bla*_CTX-M-65_, *bla*_TEM-1_, *bla*_DHA-1_, *bla*_LAP-2_	32 (R)	1 (S)	4 (R)	>64 (R)	2(S)	–		–	–	KPC+
12	2020/04	AB	KPC-76	G532T+TR insertion	D179Y+262V_268N dup	*bla*_SHV-12_, *bla*_CTX-M-65_, *bla*_TEM-1_, *bla*_DHA-1_, *bla*_LAP-2_	32 (R)	2 (I)	4 (R)	>64 (R)	2(S)	–		–	–	KPC+
13	2020/04	UR	KPC-76	G532T+TR insertion	D179Y+262V_268N dup	*bla*_SHV-12_, *bla*_CTX-M-65_, *bla*_TEM-1_, *bla*_DHA-1_, *bla*_LAP-2_	32 (R)	2 (I)	4 (R)	>64 (R)	2(S)	–		–	–	KPC+
14	2020/04	SP	KPC-76	G532T+TR insertion	D179Y+262V_268N dup	*bla*_SHV-12_, *bla*_CTX-M-65_, *bla*_TEM-1_, *bla*_DHA-1_, *bla*_LAP-2_	32 (R)	0.5 (S)	1 (S)	>64 (R)	2(S)	–		–	–	KPC+
15	2020/01	AB	KPC-76	G532T+TR insertion	D179Y+262V_268N dup	*bla*_SHV-12_, *bla*_CTX-M-65_, *bla*_TEM-1_, *bla*_DHA-1_, *bla*_LAP-2_	32 (R)	1 (S)	4 (R)	>64 (R)	2(S)	–		–	–	KPC+
16	2019/10	SP	KPC-76	G532T+TR insertion	D179Y+262V_268N dup	*bla*_SHV-12_, *bla*_CTX-M-65_, *bla*_TEM-1_, *bla*_DHA-1_, *bla*_LAP-2_	32 (R)	2 (I)	4 (R)	>64 (R)	2(S)	–		–	–	KPC+
17	2020/05	SP	KPC-76	G532T+TR insertion	D179Y+262V_268N dup	*bla*_SHV-12_, *bla*_CTX-M-65_, *bla*_TEM-1_, *bla*_DHA-1_, *bla*_LAP-2_	32 (R)	1 (S)	2 (I)	>64 (R)	2(S)	–		–	–	KPC+
18	2020/09	SF	KPC-78	A533C	D179A	*bla*_SHV-12_, *bla*_CTX-M-65_, *bla*_TEM-1_, *bla*_DHA-1_, *bla*_LAP-2_	32 (R)	0.5 (S)	2 (I)	>64 (R)	1(S)	–		–	KPC+	KPC+
19	2019/09	SP	KPC-79	TR insertion	262V_268N dup	*bla*_SHV-12_, *bla*_CTX-M-65_, *bla*_TEM-1_, *bla*_DHA-1_, *bla*_LAP-2_	32 (R)	4 (R)	8 (R)	64 (R)	4(S)	Ind		–	KPC+	KPC+

a Ind, indeterminate; TR, tandem repeat; dup, duplication; R, resistant; I, intermediary; S, sensitivity; ETP, ertapenem; IPM, imipenem; MEM, meropenem; CZA, ceftazidime-avibactam; MEV, meropenem-vaborbactam; AB, abdominal fluid; UR, urine; SP, sputum; SF, cerebrospinal fluid; MB, Mini-BAL; ND, not determined; MBL, metallo-β-lactamase.

**TABLE 2:** Results of carbapenemase detection using four assays^*a*^

ATCC strain	KPC variant	Nucleotide	Amino-acid substitution	Other β-lactams genes	MIC (mg/L)					Detection method				
					ETP	IPM	MEM	CZA	MEV	mCIM	eCIM	APB/EDTA	NG-test Carba 5	GeneXpert Carba-R
BAA-1705	KPC-2	−	−	−	>32 (R)	16 (R)	32 (R)	0.5 (S)	≤0.06 (S)	+	−	Class A carbapenemase +	KPC+	KPC+
BAA-2146	NDM-1	−	−	−	>32 (R)	>32 (R)	>32 (R)	>64 (R)	>64 (R)	+	+	MBL+	NDM+	NDM+
25922	−	−	−	−	≤0.06 (S)	0.125 (S)	≤0.06 (S)	0.5 (S)	≤0.06 (S)	−	−	−	−	−

a ETP, ertapenem; IPM, imipenem; MEM, meropenem; CZA, ceftazidime-avibactam; MEV, meropenem-vaborbactam.

### Differences in four carbapenemase detection methods for detecting *bla*_KPC-2_ variants.

**FIG 1:**
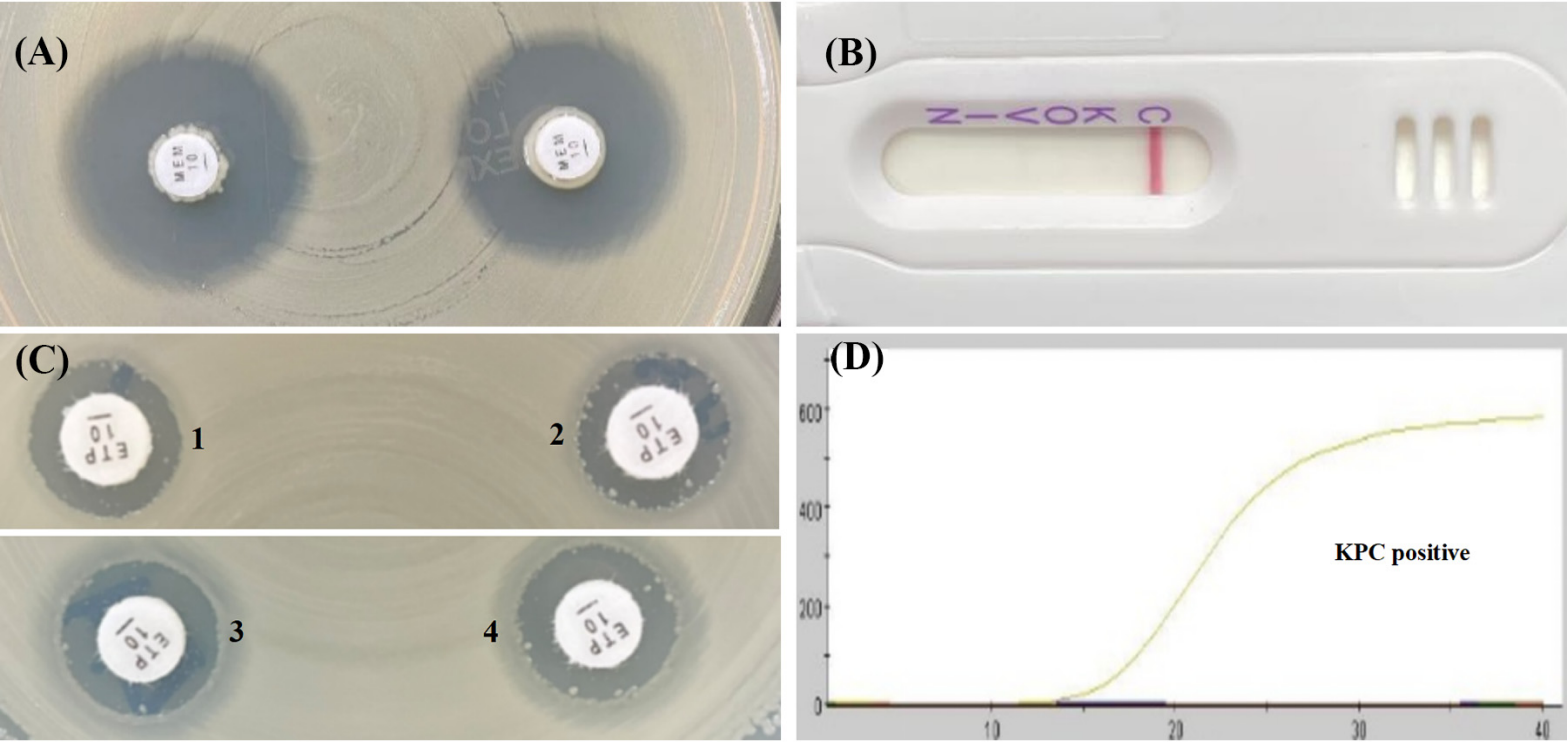
Characterization of four carbapenemase methods for detecting *bla*_KPC-33_ variant. (A) mCIM result showed a zone diameter of 22 mm, indicating carbapenemase negative. (B) APB/EDTA method, (panel B1) ertapenem (10 μg), 12 mm; (panel B2) ertapenem plus APB (300 μg), 12 mm; (panel B3) ertapenem plus EDTA (292 μg), 12 mm; (panel B4) ertapenem plus APB and EDTA, 12 mm; judged as carbapenemase negative. (C) NG-test Carba 5: KPC negative. (D) GeneXpert Carba-R: KPC positive.

**FIG 2:**
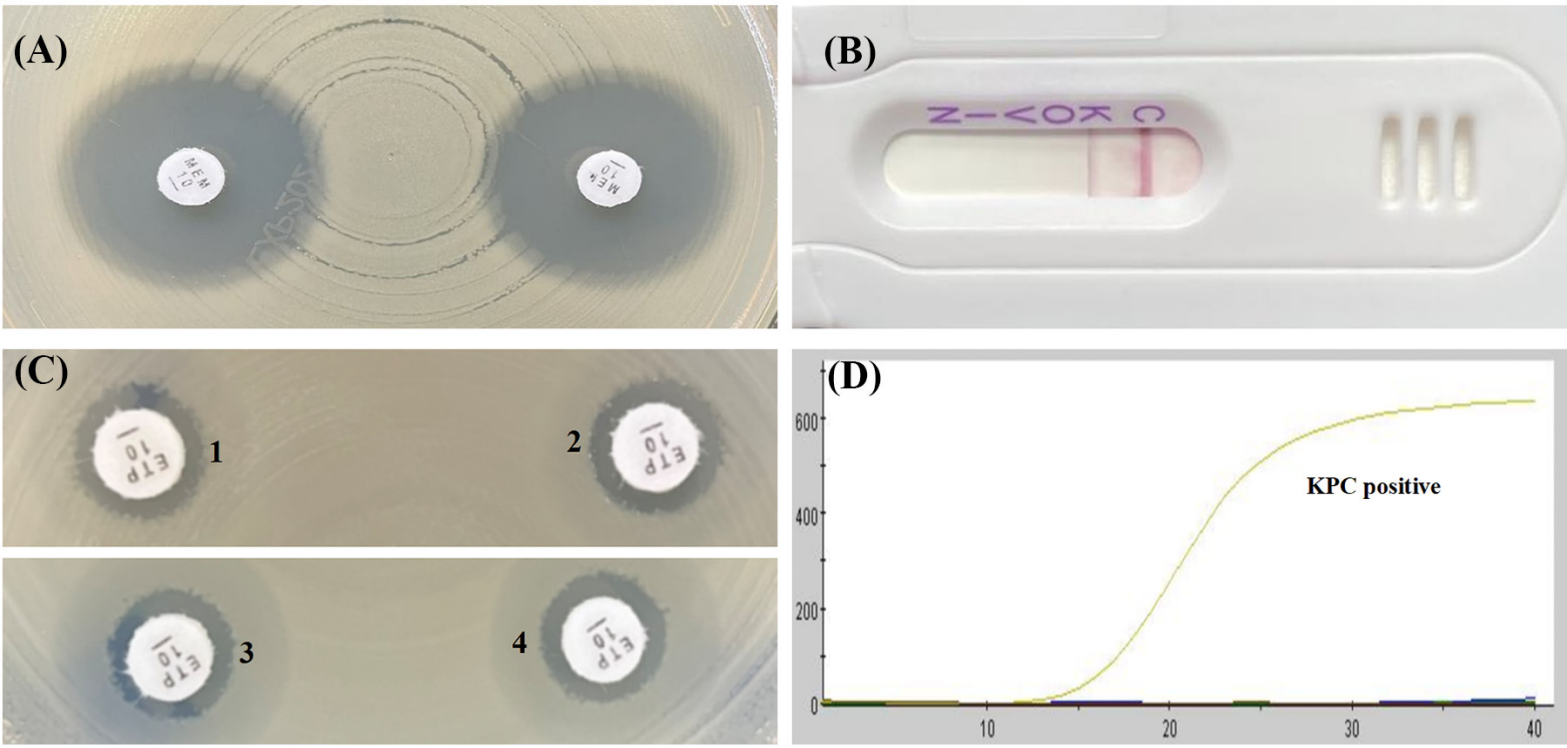
Characterization of four carbapenemase methods for detecting *bla*_KPC-35_ variant. (A) mCIM result showed a zone diameter of 22 mm, indicating carbapenemase negative. (B) APB/EDTA method, (panel B1) ertapenem (10 μg), 11 mm; (panel B2) ertapenem plus APB (300 μg), 11 mm, (panel B3) ertapenem plus EDTA (292 μg), 10 mm; (panel B4) ertapenem plus APB and EDTA, 11 mm; judged as carbapenemase negative. (C) NG-test Carba 5: KPC positive. (D) GeneXpert Carba-R: KPC positive.

**FIG 3:**
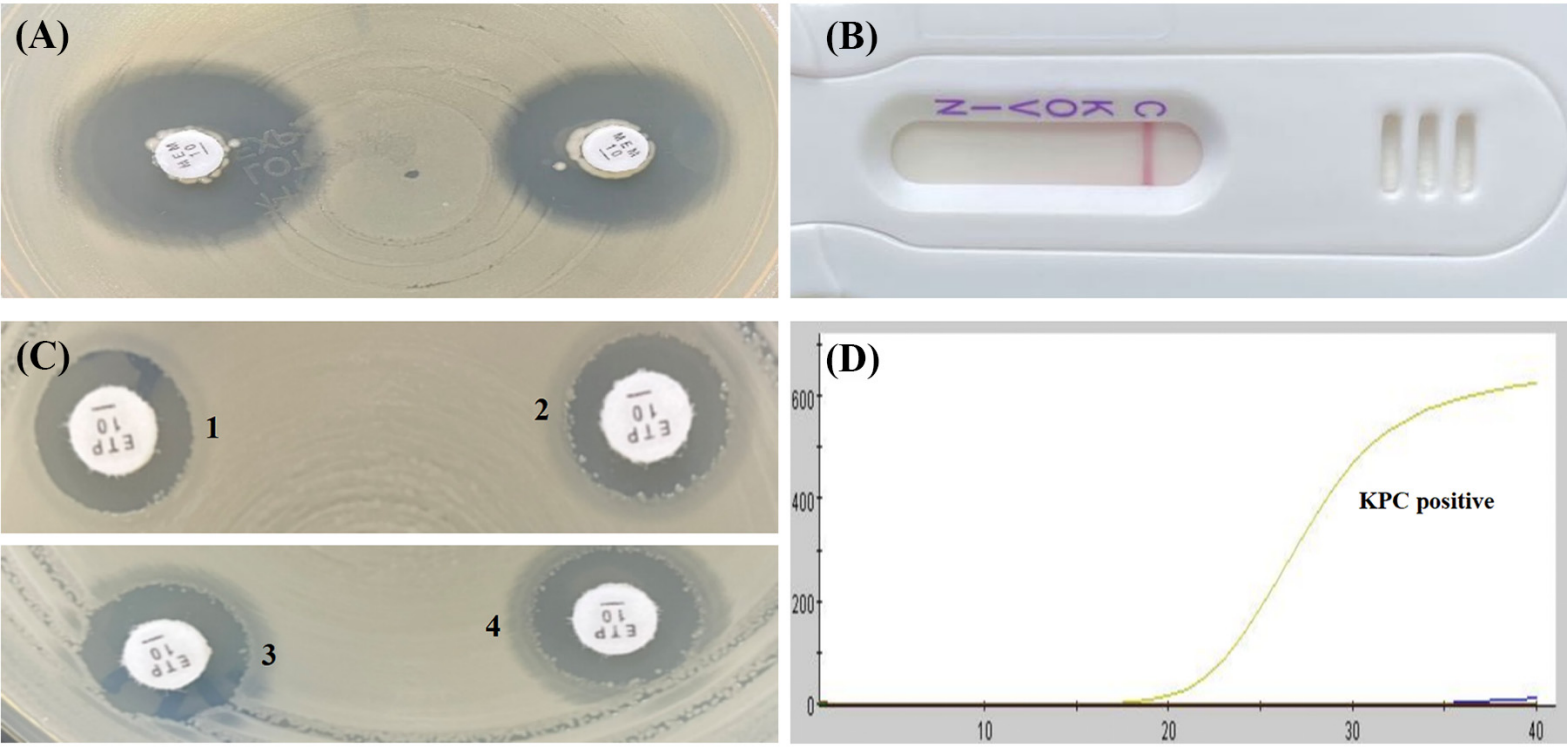
Characterization of four carbapenemase methods for detecting *bla*_KPC-71_ variant. (A) mCIM result showed a zone diameter of 21 mm, indicating carbapenemase negative. (B) APB/EDTA method (panel B1) ertapenem (10 μg), 11 mm; (panel B2) ertapenem plus APB (300 μg), 11 mm; (panel B3) ertapenem plus EDTA (292 μg), 12 mm; (panel B4) ertapenem plus APB and EDTA, 12 mm; judged as carbapenemase negative. (C) NG-test Carba 5: KPC negative. (D) GeneXpert Carba-R: KPC positive.

**FIG 4:**
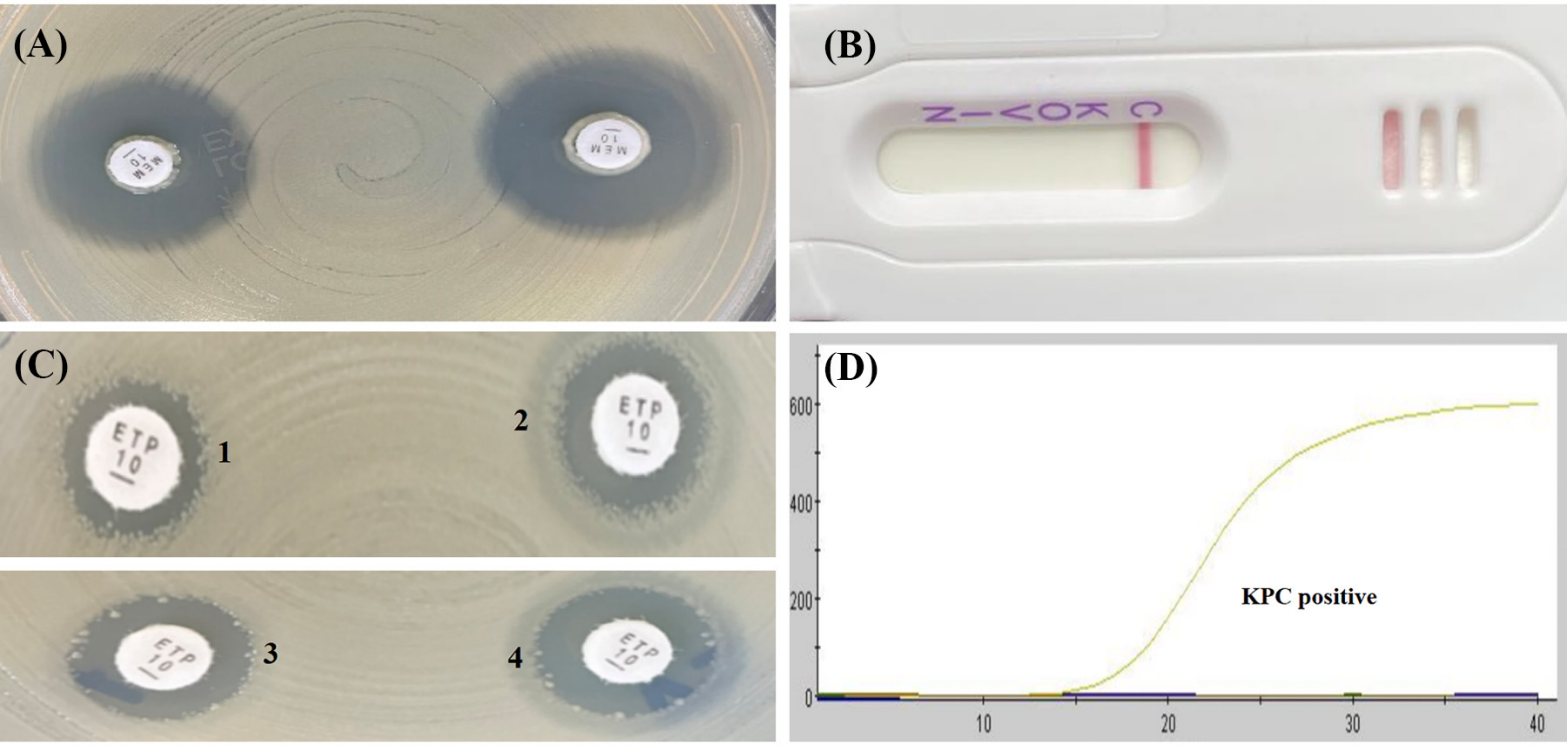
Characterization of four carbapenemase methods for detecting *bla*_KPC-76_ variant. (A) mCIM result showed a zone diameter of 22 mm, indicating carbapenemase negative. (B) APB/EDTA method, (panel B1) ertapenem (10 μg), 10 mm; (panel B2) ertapenem plus APB (300 μg), 12 mm; (panel B3) ertapenem plus EDTA (292 μg), 12 mm; (panel B4) ertapenem plus APB and EDTA, 13 mm; judged as carbapenemase negative. (C) NG-test Carba 5:negative. (D) GeneXpert Carba-R: KPC positive.

**FIG 5:**
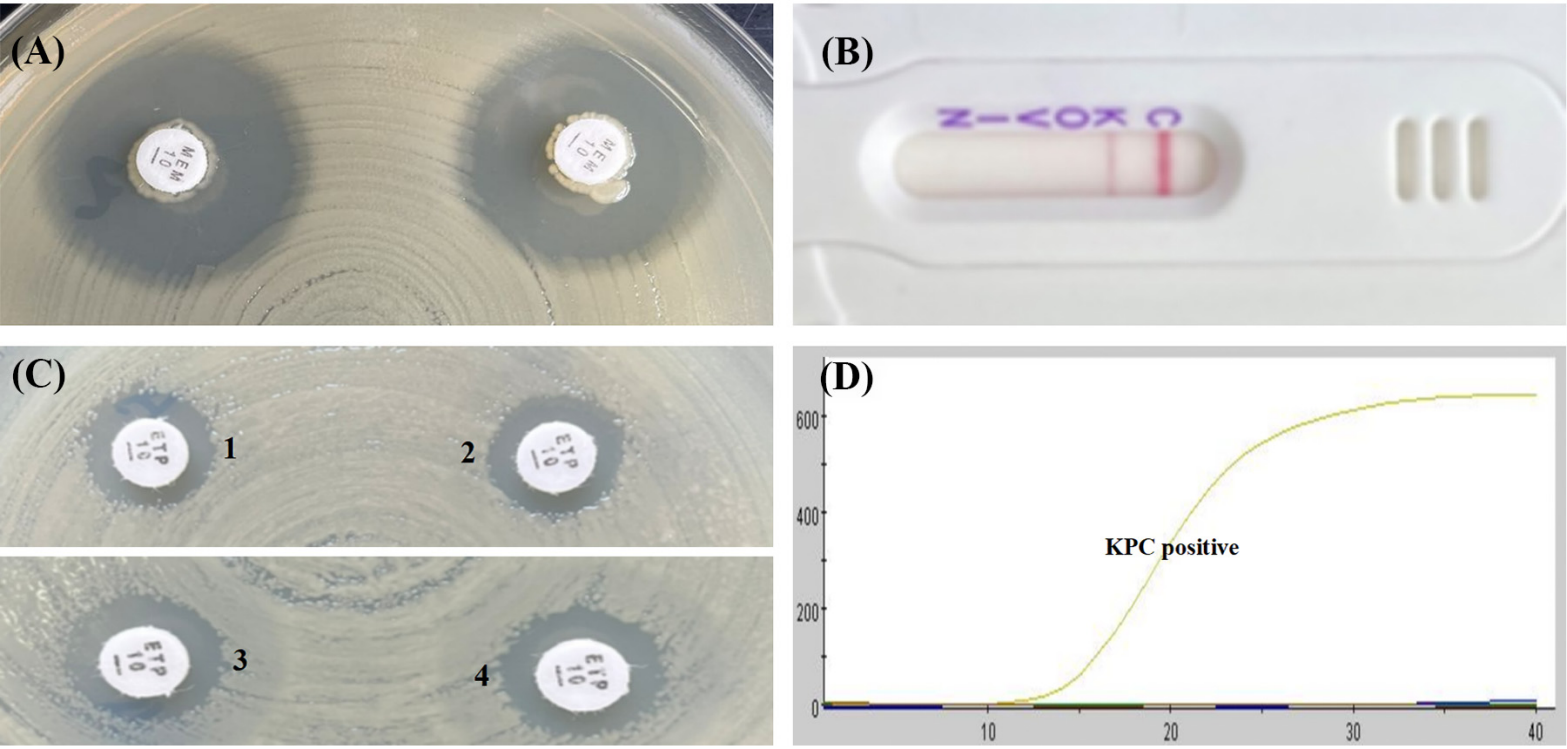
Characterization of four carbapenemase methods for detecting *bla*_KPC-78_ variant. (A) mCIM result showed a zone diameter of 21 mm, indicating carbapenemase negative. (B) APB/EDTA method, (panel B1) ertapenem (10 μg), 11 mm; (panel B2) ertapenem plus APB (300 μg), 11 mm; (panel B3) ertapenem plus EDTA (292 μg), 11 mm; (panel B4) ertapenem plus APB and EDTA, 11 mm; judged as carbapenemase negative. (C) NG-test Carba 5: KPC positive. (D) GeneXpert Carba-R: KPC positive.

**FIG 6:**
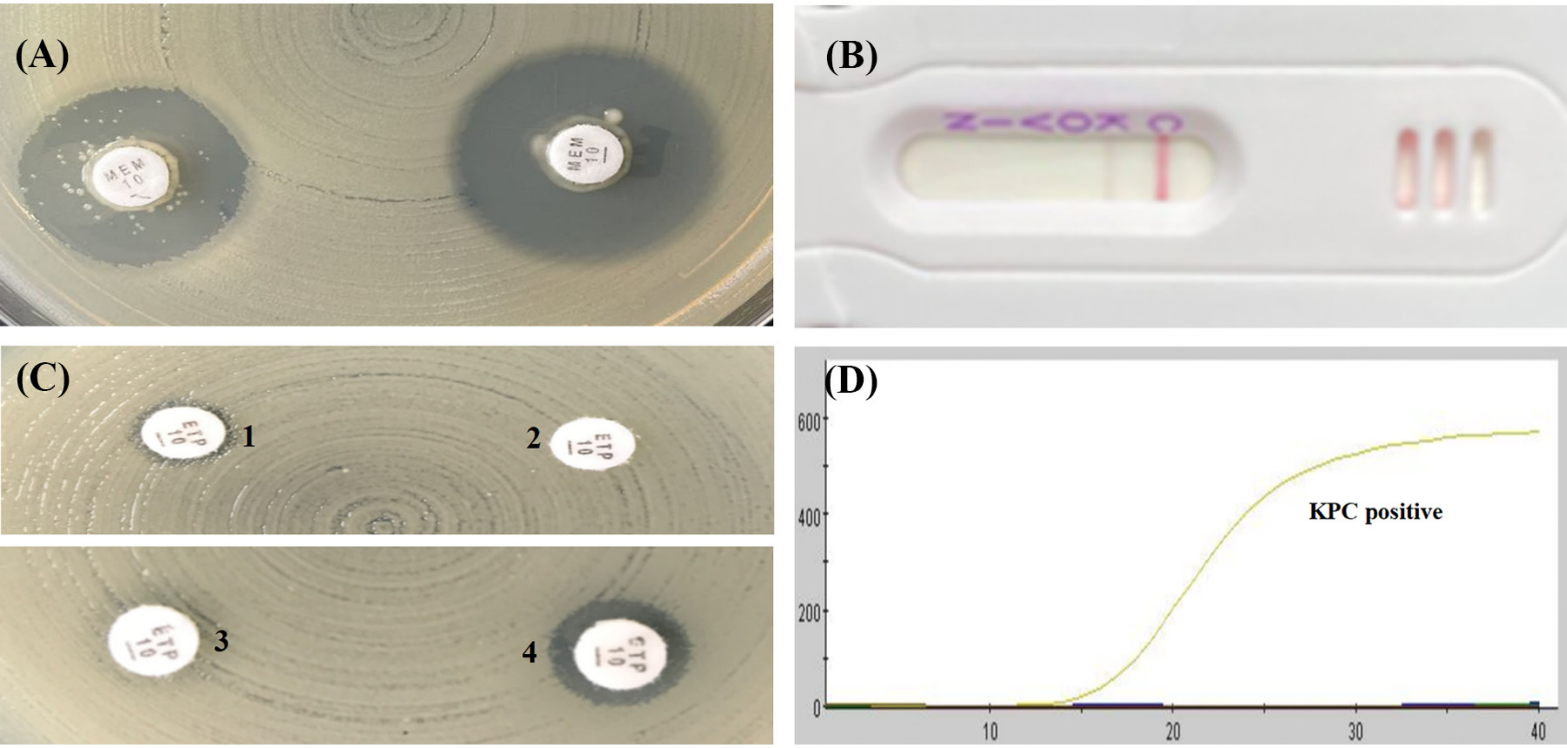
Characterization of four carbapenemase methods for detecting *bla*_KPC-79_ variant. (A) mCIM result showed a zone diameter of 19 mm and the presence of pinpoint colonies within the zone, which was interpreted as carbapenemase indeterminate. (B) APB/EDTA method, (panel B1) ertapenem (10 μg), 6 mm; (panel B2) ertapenem plus APB (300 μg), 6 mm; (panel B3) ertapenem plus EDTA (292 μg), 6 mm; (panel B4) ertapenem plus APB and EDTA, 8 mm; judged as carbapenemase negative. (C) NG-test Carba 5: KPC positive. (D) GeneXpert Carba-R: KPC positive.

As shown in Table 1 and Table 2, the mCIM results showed that the inhibition zone diameters of the 18 strains were all >19 mm, and were judged as carbapenemase negative (Fig. 1A, 2A, 3A, 4A, and 5A).The one remaining strain (harboring *bla*_KPC-79_) showed a zone diameter of 19 mm and pinpoint colonies within the zone; this was interpreted as carbapenemase indeterminate, meaning that the presence or absence of carbapenemase could not be confirmed (Fig. 6A). The eCIM results for the clinical strains were meaningless because eCIM was performed only when the mCIM test was positive. For the APB/EDTA method results, all 19 strains carrying the *bla*_KPC-2_ variant showed a <5 mm increase in the zone diameters of the combined disks, e.g., ertapenem with APB and/or EDTA, compared with the zone diameters for ertapenem alone, and they were judged as carbapenemase negative (Table 1). Interestingly, the NG-test Carba-5 results showed that five strains (harboring *bla*_KPC-35_, *bla*_KPC-78_, and *bla*_KPC-79_) were KPC positive (Fig. 2C, 5C, and 6C, respectively), and the remaining 14 strains (harboring *bla*_KPC-33_, *bla*_KPC-71_, and *bla*_KPC-76_) showed negative results (Fig. 1C, 3C, and 4C, respectively). Besides this, the GeneXpert Carba-R results showed that all 19 strains were *bla*_KPC_ positive (Table 1).

## DISCUSSION

Since different antimicrobial agents have varying antimicrobial activities against different carbapenemase-producing strains *in vitro*, the accurate and rapid detection of carbapenemase is of great value for precise dosing in clinical anti-infective therapy, hospital infection prevention, and control of carbapenemase-producing *Enterobacterales* clinical isolates. For example, ceftazidime-avibactam has excellent antibacterial activity against KPC and OXA-48 serine carbapenemase-producing strains but no activity against MBL-producing strains (10). Nonetheless, more and more studies have reported that *Enterobacterales* with *bla*_KPC-2_ and *bla*_KPC-3_ variants are resistant to ceftazidime-avibactam, leaving less effective treatment options available. In addition, meropenem-vaborbactam has high antibacterial activity against KPC-producing strains (including KPC-2 and KPC-3 variants), but no antibacterial activity against MBL-producing and OXA-48 carbapenemase strains (9).

Several *bla*_KPC-2_ variants display a decrease or loss of carbapenemase activity *in vitro*, as was seen in several of our isolates. Their resistance to ceftazidime-avibactam has led to widespread interest in *bla*_KPC_ variants. Recently, Bianco et al. (18) highlighted some important limitations of the main carbapenemase detection methods (including NG-test Carba 5, RESIST-5 O.O.K.N.V, Rapidec Carba NP, mCIM, and the disk diffusion synergy test) in detecting some *bla*_KPC_ variants (including *bla*_KPC-14,_ *bla*_KPC-33_, and *bla*_KPC-31_) associated with ceftazidime-avibactam resistance during therapy. Likewise, we confirmed that some *bla*_KPC-2_ variants (including *bla*_KPC-33_, *bla*_KPC-35_, *bla*_KPC-71_, *bla*_KPC-76_, *bla*_KPC-78_, and *bla*_KPC-79_) could not be detected by mCIM or the APB/EDTA method, which may be related to the mutation of *bla*_KPC-2_ leading to a loss of carbapenemase activity. Of these identified mutations, *bla*_KPC-33_, *bla*_KPC-35_, and *bla*_KPC-78_ comprised single-nucleotide substitutions (G to T, T to C, and A to C), which caused amino acid changes at positions 179 (D179Y), 169 (L169P), and 179 (D179A), respectively. One or more amino acid insertions were identified in *bla*_KPC-71_, *bla*_KPC-76_, and *bla*_KPC-79_ (Table 1). Alternatively, the different results of NG-test Carba 5 appear to be related to the location where the variant occurs in the *bla*_KPC-2_ gene. Of the negative-resultant *bla*_KPC-2_ variants (including *bla*_KPC-33_, *bla*_KPC-71,_ and *bla*_KPC-76_), the *bla*_KPC-33_ and *bla*_KPC-76_ variants had identical amino acid changes at position 179 (D179Y), and the *bla*_KPC-71_ variant had an amino acid insertion at position 181. Although KPC-78 can be detected by NG-test Carba 5, it has a very light-colored band. These findings suggested that positions 179 or the surrounding amino acids in KPC are critical for correct enzyme immunodetection by LFIA. In contrast, the GeneXpert Carba-R assay is based on qPCR, and its results are not affected by gene mutations. Therefore, laboratories cannot rely solely on carbapenemase detection to speculate on a strain’s susceptibility to ceftazidime-avibactam. Rather, they should simultaneously perform ceftazidime-avibactam susceptibility to better guide clinical anti-infective therapy.

But there are some limitations to this study. First of all, our sample size is limited, with only six subtypes of *bla*_KPC-2_ variants (including *bla*_KPC-33_, *bla*_KPC-35_, *bla*_KPC-71_, *bla*_KPC-76_, *bla*_KPC-78_, and *bla*_KPC-79_) collected in our hospital. Second, these variants are all derived from *bla*_KPC-2_ mutations, with none derived from *bla*_KPC-3_ mutations. Therefore, it is not possible to evaluate the differences between these four methods for detecting *bla*_KPC-3_ variants.

The results of antimicrobial susceptibility testing in this study indicated that the isolates carrying KPC variants acquired by mutations in *bla*_KPC-2_ were usually susceptible to imipenem but resistant to meropenem and ertapenem. These means that the KPC variants have not lost all carbapenemase activity *in vitro*. Therefore, it is clinically necessary to identify them as carbapenemase-producers, and infected patients still need to be strictly managed. Although the isolates with KPC variants may have increased susceptibility to imipenem, available study data suggest that the *bla*_KPC-2_-positive strain dominated again following imipenem substitution therapy for the ceftazidime-avibactam resistant *bla*_KPC-33_ variant (11). In addition, ceftazidime-avibactam-resistant strains emerge as resistant to meropenem during *in vitro* passage at subinhibitory meropenem concentrations, so the role of carbapenems in treating patients with such bacterial infections is unclear (19). Thus, clinical laboratories must seek suitable detection methods for the identification of these strains in order to avoid nosocomial transmission.

Furthermore, laboratories encountering strains which have specific resistance phenotypes (e.g., a strain harboring *bla*_KPC-33_ shows resistance to ceftazidime-avibactam but sensitivity to imipenem) and test negative for carbapenemase when using conventional methods should further define the resistance mechanism by using sequencing to identify possible genetic subtypes. In the meantime, existing assays should be continuously improved for better detection of new KPC variants.

## MATERIALS AND METHODS

### Clinical strains.

Nineteen K. pneumoniae carrying *bla*_KPC-2_ variants were collected at Huashan Hospital (Shanghai, China) during 2019 to 2021, isolated from sputum, urine, abdominal fluid, and cerebrospinal fluid. Species identification was confirmed by a MALDI-TOF/MS system (bioMérieux, France). The presence and subtypes of the *bla*_KPC-2_ gene were initially confirmed by PCR-based DNA sequencing, including whole-genome sequencing using Illumina (Illumina, San Diego, CA, USA), and were compared with available sequences in GenBank. An additional antimicrobial resistance gene analysis was performed using ResFinder 4.1 (https://cge.cbs.dtu.dk/services/ResFinder/). Among those *bla*_KPC-2_ variants, *bla*_KPC-33_-, *bla*_KPC-35_-, *bla*_KPC-71_-, *bla*_KPC-76_-, *bla*_KPC-78_-, and *bla*_KPC-79_-positive isolates accounted for 26.3% (5/19), 15.8% (3/19), 5.3% (1/19), 42.1% (8/19), 5.3% (1/19), and 5.3% (1/19), respectively. K. pneumoniae ATCC BAA-1705, K. pneumoniae ATCC BAA-2146, *E.coli* ATCC 25922, respectively, were tested as a KPC-positive strain, NDM-positive strain, and carbapenemase-negative strain for quality control in carbapenemase detection. In addition, *E.coli* ATCC 25922 was included for a quality control assessment in antimicrobial susceptibility testing.

### Antimicrobial susceptibility testing.

MICs were determined by the broth microdilution method recommended by the Clinical and Laboratory Standards Institute (CLSI) with CLSI-recommended MIC breakpoints (20). Ertapenem, imipenem, meropenem, ceftazidime-avibactam, and meropenem-vaborbactam were tested in this study.

### Carbapenemase detection.

Among isolates carrying the *bla*_KPC-2_ gene variant, carbapenemase production was tested in duplicate using two phenotypic methods (mCIM/eCIM and APB/EDTA method), the LFIA NG-test Carba 5 assay, and the qPCR-based GeneXpert Carba-R assay. The phenotypic mCIM and eCIM were performed following CLSI recommended guidelines to examine whether strains could hydrolyze carbapenems (20). Briefly, a 1-μL loopful of overnight-cultured bacteria was emulsified in 2 mL tryptic soy broth (TSB). Next, a 10-μg meropenem disk was added to the bacterial suspension after vortexing for 10 s, followed by incubation at 37°C for 4 h. After the incubation time, the meropenem disk was placed on Muller-Hinton agar (MHA) which had been previously inoculated with 0.5 McFarland standard E. coli ATCC 25922 as an indicator organism. The result was evaluated by measuring the inhibition zone around the meropenem disk after incubation at 37°C for 18 to 24 h and classifying it as positive, negative, or indeterminate. For each isolate, a second 2-mL TSB tube was labeled for the eCIM test. Twenty μL of 0.5 M EDTA was added to the 2-mL TSB tube to obtain a final concentration of 5 mM EDTA. After this, the same steps as described above for the mCIM procedure were followed. The meropenem disks from the mCIM and eCIM tubes were placed on the same MHA plate inoculated with the meropenem-susceptible E. coli ATCC 25922 indicator strain. It is worth noting that eCIM was evaluated only when the mCIM test was positive. The APB/EDTA method, employing combined-disk tests of ertapenem alone, ertapenem with either 300 μg APB or 292 μg EDTA, and ertapenem with both 300 μg APB and 292 μg EDTA, was performed to detect carbapenemase production and the differentiation of class A carbapenemase and metallo-β-lactamase (MBL), as previously described (21, 22). Production of class A carbapenemase was considered positive when the growth-inhibitory zone diameters around the ertapenem disk with APB and around the ertapenem disk with both APB and EDTA was increased by ≥5 mm compared with the growth-inhibitory zone diameter around the ertapenem disk alone. Production of MBL was considered positive when the growth-inhibitory zone diameters around the ertapenem disk with EDTA and around the ertapenem disk with both APB and EDTA was increased by ≥5 mm compared with the growth-inhibitory zone diameter around the ertapenem disk alone. Additionally, the bacteria were considered negative for both class A carbapenemase and MBL production if none of the three combined-disk tests were positive (21, 22). According to the manufacturer’s instructions, a NG-test Carba 5 assay (NG Biotech, France) was used to test for KPC variants (23). Briefly, a 1-μL loopful of bacteria was mixed with five drops of Carba-5 extraction buffer. Next, 100 μL of the mixture was transferred into the Carba-5 cassette after vortexing, and the results were evaluated after incubation for 15 min (24). A GeneXpert Carba-R assay (Cepheid Inc., USA) was used to detect those KPC variants. A 10-μL suspension of 0.5 McFarland standard harvested from overnight-cultured bacteria was mixed with sample reagent in the Xpert Carba-R assay sample reagent vial. The recommended volume was added to the Xpert Carba-R cartridge with a disposable transfer pipette, and run on the GeneXpert IV system (24).

The study protocol was approved by the Institutional Review Board of Huashan Hospital, Fudan University (No.2018-408).

### Data availability.

The genome sequencing data are publicly available at NCBI GenBank under the BioProject accession number PRJNA785420.
